# E pluribus plurima: Multidimensional indices and clinical phenotypes in COPD

**DOI:** 10.1186/1465-9921-12-152

**Published:** 2011-11-14

**Authors:** Andrea Rossi, Erika Zanardi

**Affiliations:** 1 Pulmonary Unit, Cardiovascular and Thoracic Department, University and General Hospital, P.le Stefani 1, I-37126, Verona, Italy

## 

Chronic Obstructive Pulmonary Disease (COPD) is a disorder of the respiratory system characterized by progressive and only partially reversible airflow obstruction, due to a varying combination of large (bronchitis) and small airways (small airway disease) damage, and lung parenchymal and vascular destruction [1]. We prefer the term obstruction to airflow *limitation *because the latter is a physiologic event which occurs also in normals at high levels of ventilation, for example during exercise. The correct definition should be "excessive airflow limitation" to indicate that the reduction in airflow occurs at lower level of ventilation than in normal condition. The diagnostic procedure for COPD starts from the recognition of risk factors (cigarette smoking *"in primis"*, but also outdoor and indoor air pollution [2]) and the presence of symptoms such as chronic cough and phlegm and reduced exercise tolerance. The lifestyle is important for the reveal of symptoms: dyspnea occurs later in a sedentary person than in an active individual.

The objective demonstration of airflow obstruction by spirometry is mandatory to establish the diagnosis. A post-bronchodilator FEV1/FVC < 0.70 is considered sufficient to define airflow obstruction and to confirm the diagnosis [3,4]. FVC = forced vital capacity; FEV1 = forced expiratory volume in the 1st second; VC = (slow) vital capacity. Some Guidelines requires that FEV1/FVC < 0.70 [5] should be associated with a FEV1 < 80% of the predicted value [1,6]. Many Authors [7], however, and some official documents [8,9] do not accept the fixed "cut-off" and indicate the FEV1/VC < lln (lower limit of normality) as a more correct documentation of airflow obstruction. The debate is still ongoing [10]. However, it seems to be a general agreement to use the value of FEV1%predicted to stage the severity of the disease. Neverthless, it would be more appropriate to accept the use of that measurement for staging only the severity of airflow obstruction, not the whole disease state. In fact COPD is a heterogeneous disorder with diverse pathophysiological manifestations at the level of the respiratory system as well as at systemic level with complications and comorbidities. Not surprisingly, the FEV1 is rather insufficient to assess the status and progress of the disease as well as the effects of therapies. Although very helpful and valuable, the FEV1 has several limitations which should be taken into account when interpreting its value and changes.

First of all it should be remembered that FEV1 results from two undisclosed determinants, i.e. the caliber of the large airways and the lung elastic recoil. The latter is poorly sensitive to treatments whereas the former can be improved by either pharmacological [11] and/or non pharmacological [12] treatments. Therefore, the individual response to therapies depends upon which determinant drives the FEV1 reduction more. Furthermore, the FEV1 is rather insensitive to small airway disease, which is an important pathology of COPD [13] and may be extensively present when spirometry is still within the normal range [14].

### Respiratory pathophysiology

Airflow obstruction is the hallmark of COPD. However, the pathophysiology of COPD is intricate. In fact, it encompasses also pulmonary hyperinflation and nonuniform distribution of ventilation [15]. Lung hyperinflation has two components:

*• static*, i.e. the increase in functional residual capacity (FRC) due to the loss of lung elastic recoil because of destruction of lung parenchyma, and

*• dynamic*, i.e. the position of the end-expiratory lung volume above the relaxed volume of the respiratory system, (for example during exercise or exacerbations).

Hyperinflation may be a predictor of mortality when expressed as IC/TLC%. IC = inspiratory capacity; TLC = total lung capacity [16].

Small airways disease and parenchymal destruction result in maldistribution of ventilation leading to ventilation-perfusion mismatching and eventually causing lung failure and hypoxemia. On the other hand, pulmonary hyperinflation reduces the pressure generating capacity of the respiratory muscles eventually leading to ventilatory pump failure, and hypercapnia [17]. None of these pathophysiologic events is correlated to the changes in FEV1, which, at the same time, is poorly related to exercise capacity and symptoms intensity. However, this elaborate respiratory pathophysiology is not the end of the COPD heterogeneous picture. Systemic effects must be taken into account to understand correctly the real severity of the disease in different patients.

### Systemic effects

The skeletal muscles are affected unfavourably by COPD. Exercise intolerance worsens with the progression of the disease [18]. Obviously, the first individual reaction is to prevent that "unpleasant sensation of difficult breathing" (i.e. dyspnea) by limiting exercise and life activity. Under those circumstances, skeletal muscles undergo progressive deconditioning and the vicious circle is elicited: dyspnea - activity limitation - muscle deconditioning - dyspnea [19]. Often, malnutrition can aggravate the loss of skeletal muscles force.

Chronic cor pulmonale is a well known complication of advanced COPD. However, a recent large, population based study has shown that impaired left ventricular filling, reduced stroke volume, and lower cardiac output were linearly related to the extent of emphysema at the CT scanning and to the severity of spirometric airflow obstruction [20]. However, in that study, the FEV1/FVC ratio was, on average, above 0.64, a value only slightly below the 0.70 limit accepted as normal. Therefore, the cardiovascular system in COPD patients is challenged not only by the common risk factor, i.e. cigarette smoking, but also by emphysema at earlier stages than traditionally thought.

### Exacerbations

The assessment of COPD severity cannot ignore the occurrence of exacerbations, which are a prominent feature of the natural history of COPD. They influence the progression of the disease and are a major cause of morbidity and mortality, and socio-economic cost [21]. Many data support the conclusion that exacerbations are more frequent and more severe in patients with advanced airflow obstruction. However, the ECLIPSE study [22] found that although exacerbations become more frequent and more severe as COPD progresses, the rate at which they occur appears to reflect an independent susceptibility phenotype.

Therefore, it is not surprising that a single variable, such as for example the FEV1, cannot capture the heterogeneity of COPD, both pulmonary and systemic [23]. In addition, the individual patient with COPD is often affected by comorbidities, because other diseases are common at the age when COPD becomes clinically revealed, for example systemic arterial hypertension, diabetes, obesity etc.

In summary, the modern approach to the COPD patient goes beyond the necessary demonstration of airflow obstruction and the understanding of the complex pulmonary pathophysiology to embody the systemic effects and comorbidities [24,25]. This view encouraged the development of multidimensional indices to provide physician with some robust instrument to ascertain the status and progress of the disease as well as to guide therapy in the individual patient.

In this issue of Respiratory Research, Wouter D. van Dijk and colleagues [26] provide a systematic review of 15 multidimansional indices selected in 13 studies from > 7000 articles screened in the Pubmed and Embase literature database. This laudable effort concluded however that "although the prognostic performance of the indices has been validated, they all lack sufficient evidence on implementation". Obviously it is not an Authors' fault. It is the discrepancy between the complexity of the disease and the need to find something measurable to be helpful, clear, and easy for its use in the clinical practice. The population based prediction might be improved by some indices which may however lack feasibility in the real life of the individual patient-doctor relationship. A good example of this dilemma is the FEV1 decline. Since the classic study by Fletcher and colleagues [27,28] it is widely accepted that the rate of decline of the FEV1 might be regarded as a marker of the progression of COPD related to important outcomes such as disability and death [29]. However, the starting point of the FEV1 might be influenced by the impact of factors, not related to COPD, in early life or even in the pre-birth period on adult lung function. Furthermore, the rate of decline of the FEV1 cannot be used in the clinical practice because it would require a minimum of observation for two years, with at least three-four measurements of FEV1 per year: the first year to compute the baseline decay and the second year, after the start of the treatment, to document the slowdown of the decay. Rather impractical!

Great expectations are generated by molecular and genomic research [30]. The result of gene-environment interactions determines the clinical presentation of the disease: the *clinical phenotype*. It might be that a better identification of COPD phenotypes would lead to identification of specific indices customized to a particular phenotype. The traditional classification of COPD phenotypes pertains to the classic "blue-bloater" and "pink-puffer" pictures [31]. However, it has been suggested already several years ago that many patients fall into neither group and that those descriptive terms are not clearly related to specific functional or pathologic features. Therefore its use is not encouraged [8]. By contrast, other phenotypes have been recommended. The ECLIPSE study suggests the "frequent exacerbator" phenotype [32-34], which could be further classified into three "clinical phenotypes" termed bacteria-associeted, virus-associeted and eosinophil-associeted [34]. This has meaningful implication for clinical practice for both the therapeutic approach [21,35] and the choice of the multidimensional index, which should include the exacerbation occurrence. For example, the DOSE [36] might apply better to this phenotype rather than to the general COPD population.

Currently, COPD and asthma are differentiated, but we all accept that some areas of overlap exist. Their recognition may influence the therapeutic decision, for example the use of inhaled corticosteroids [3,37,38]. Lung function tests such as assessment of airway reactivity [39] or measurement of single-breath carbon monoxide transfer factor (TL,CO) [8,40] could be particularly useful to monitor this segment of patients. In some cases the detection of eosinophilic sputum might be useful [41].

The analysis of data from large, longitudinal studies has brought to attention the fact that FEV1 decline is not uniform throughout the progression of the disease but it may be larger at early stage, when there is more to lose, and smaller in the advanced stage when it remains little to be lost [42,43]. A subgroup of so called "rapid decliner" [44,45] might reflect another phenotype of the disease. In this subgroup, or clinical phenotype, repeated measurement of FEV1 could be much more valuable than in other sub-groups.

In conclusion, as the picture of COPD becomes more complex and the results from large studies generate the need of further research, it is clear the close link between the definition of clinical phenotypes and the validation of either single or multidimensional indices. The line of search marker, either biological or physiological, for one COPD has come to its end. The definition of multiple clinical phenotype crosses repeatedly and systematically the evolution of indices and markers. From the cross-matching of multiple phenotypes and multidimensional indices we cannot expect the birth of a single variable to assess the heterogeneous COPD, but multiple variables for different COPDs: "*e pluribus plurima"*.
